# The classification of absence seizures using power-to-power cross-frequency coupling analysis with a deep learning network

**DOI:** 10.3389/fninf.2025.1513661

**Published:** 2025-02-10

**Authors:** A.V. Medvedev, B. Lehmann

**Affiliations:** EEG and Optical Imaging Laboratory, Center for Functional and Molecular Imaging, Georgetown University Medical Center, Washington, DC, United States

**Keywords:** absence seizure, epilepsy, seizure classification, EEG, spectral analysis, cross-frequency coupling (CFC), power-to-power coupling

## Abstract

High frequency oscillations are important novel biomarkers of epileptic tissue. The interaction of oscillations across different time scales is revealed as cross-frequency coupling (CFC) representing a high-order structure in the functional organization of brain rhythms. Power-to-power coupling (PPC) is one form of coupling with significant research attesting to its neurobiological significance as well as its computational efficiency, yet has been hitherto unexplored within seizure classification literature. New artificial intelligence methods such as deep learning neural networks can provide powerful tools for automated analysis of EEG. Here we present a Stacked Sparse Autoencoder (SSAE) trained to classify absence seizure activity based on this important form of cross-frequency patterns within scalp EEG. The analysis is done on the EEG records from the Temple University Hospital database. Absence seizures (*n* = 94) from 12 patients were taken into analysis along with segments of background activity. Power-to-power coupling was calculated between all frequencies 2–120 Hz pairwise using the EEGLAB toolbox. The resulting CFC matrices were used as training or testing inputs to the autoencoder. The trained network was able to recognize background and seizure segments (not used in training) with a sensitivity of 93.1%, specificity of 99.5% and overall accuracy of 96.8%. The results provide evidence both for (1) the relevance of PPC for seizure classification, as well as (2) the efficacy of an approach combining PPC with SSAE neural networks for automated classification of absence seizures within scalp EEG.

## 1 Introduction

Brain oscillations span frequencies across a range of several orders of magnitude from the Berger bands below 30 Hz (delta, theta, alpha, beta) up to the high frequency bands of gamma, ripple, and fast ripple (30–600 Hz). This study was inspired by emerging evidence that brain oscillations do not work independently from each other but interact in a very complex and well-coordinated way known as cross-frequency coupling (CFC) (Buzsaki and Draguhn, 2004; Klimesch, 2013). Cross-frequency coupling plays an important role in the functional organization of neural networks at different spatial and temporal scales. This coupling represents a high-order structure in the functional organization of brain rhythms and is likely to reflect different functional states of the brain (Buzsaki and Draguhn, 2004). It is reasonable to suppose that optimal biomarkers of complex neurological processes would have sensitivity to this structure, going beyond isolated features (e.g., frequency or spectral characteristics).

In recent years, there has been a burgeoning interest in high-frequency oscillations (HFOs) driven by emerging evidence suggesting their involvement in cognitive functions (Gross and Gotman, 1999; Hosseinzadeh et al., 2005; Axmacher et al., 2008; Medvedev and Kanwal, 2008; Buzsaki and Silva, 2012; Kucewicz et al., 2014; Pail et al., 2020; Dickey et al., 2022). Also, heightened activity in these frequency ranges has been observed in pathological conditions and, in particular, numerous studies have demonstrated a significant increase in HFOs in the context of epilepsy. Those studies have revealed that HFOs are one of the most common early manifestations recorded within minutes before seizure onset and appear to be a reliable EEG correlate of ictal onset zone (Lee et al., 2000; Medvedev, 2002; Worrell et al., 2004; Gardner et al., 2007; Jacobs et al., 2008; Blanco et al., 2010; Medvedev et al., 2011). Several research groups have suggested that detection of HFOs is necessary for a more accurate localization of epileptogenic tissue. Improvements in accuracy may improve surgical outcome in patients with localization-related intractable epilepsy because the removal of HFO-generating areas correlates with good surgical outcomes (Bragin et al., 1999; Worrell et al., 2004; Gardner et al., 2007; Besio et al., 2010; Zijlmans et al., 2012; Staba et al., 2014; Frauscher et al., 2017; Medvedev et al., 2019; Thomschewski et al., 2019). Thus, in addition to epileptic discharges, HFOs are now considered as an important biomarker of epileptogenic tissue.

High-frequency bursts are frequently accompanied by low-frequency waveforms, such as sharp waves and spikes. These patterns may signify specific forms of cross-frequency coupling. The most typical examples pertinent to epilepsy include the Ripple-on-Spike, where a high-frequency burst is riding on a spike, as well as the Ripple-on-Oscillation, where a high-frequency burst is riding on a slow wave. Given that epileptic seizures are often accompanied by specific patterns of cross-frequency coupling between slow and fast activity, it is important to explore the possibility that cross-frequency coupling may be used as a tool for automated detection of seizures.

Absence seizures are traditionally characterized by spike-and-wave activity with the dominant frequency of 3–4.5 Hz. This specific narrow frequency range and the regular morphological features of absence seizures offer a good starting point from which to evaluate epilepsy using a new CFC approach. More specifically, these reliable characteristics of absence epilepsy in combination with research connecting HFO’s with epileptogenic tissue (Chaitanya et al., 2015) suggest the possibility of interaction between low and high frequency bands. Furthermore, approaches that can unveil these dynamic relationships may identify more comprehensive signatures of absence epilepsy (e.g., beyond describing which waveband amplitudes are merely involved). Therefore, such approaches hold promise both for optimal classification power and for advancing the understanding of the neurobiology of seizures.

Methods utilizing cross-frequency coupling have shown predictive power in various areas of EEG research including epilepsy state classification (Jacobs et al., 2018). There are various types of coupling (i.e., power-to-power, power-to-phase, phase-to-phase, etc.). These different types are thought to have independent neural mechanisms as well as different or complimentary functional significance (Jirsa and Muller, 2013). While many forms of CFC have not been well-researched, one of the better-studied forms of CFC is phase-to-amplitude coupling (PAC), which is well known to have an association with various cognitive processes related to memory and perception (Gross and Gotman, 1999; Axmacher et al., 2008; Medvedev and Kanwal, 2008; Buzsaki and Silva, 2012; Kucewicz et al., 2014; Dickey et al., 2022). In many cases, PAC refers to the phase of a slower wave modulating the amplitude of the faster wave. In regards to seizure classification, prior studies (including both EEG and intracranial EEG) have linked delta-HFO coupling with epileptogenic tissue, and have employed this feature in discriminating between ictal and interictal states (Ibrahim et al., 2014; von Ellenrieder et al., 2016; Edakawa et al., 2016). For example, Jacobs et al. (2018) used a random forest algorithm on PAC and obtained a sensitivity (*Sens*) of 87.9% and specificity (*Spec*) of 82.4% for classification of pre-clinical seizure states. More specifically, they found increases in coupling between delta (2–4 Hz) and gamma (20–50 Hz) bands to be a key feature for classifying the seizure EEG patterns (Jacobs et al., 2018). Fujita et al. (2022) using a deep learning (DL) classifier found training the network on PAC significantly improved seizure classification over training on the raw data, achieving 90% accuracy (*Acc*) using the former method (Fujita et al., 2022). It is notable that the delta-theta activity coupled with the gamma band is not strictly pathological, and is thought to be involved in working memory, sensory and other cognitive processes (Lisman and Jensen, 2013). While highly informative, PAC remains just one of many presumably complimentary forms of cross-frequency coupling that may hold keys to functional and pathological states of the brain.

Power-to-power coupling (PPC) is another type of cross-frequency coupling having a solid research base (Llinas et al., 1999; Shirvalkar et al., 2010; Popov et al., 2018; Wang et al., 2018; Sheremet et al., 2019) attesting to its significance, yet in contrast with PAC, it has a surprising lack of research in the area of seizure classification. PPC has been used for well over two decades in both murine and human studies, and across data types including local field potential (LFP), EEG and MEG. Some examples include tracking coupling between theta and gamma or other sets of frequencies within the rat hippocampus (Sheremet et al., 2019). PPC is found to be involved with successful memory retrieval (Shirvalkar et al., 2010) and other PPC patterns have been associated with specific states including sleep and anesthesia (Ferraris et al., 2018). While there is a sound foundation of research attesting to the value of PPC for identifying biomarkers, it has not been researched in the area of seizure classification.

Power-to-power coupling should be particularly amenable to long-term monitoring of patients due to its methodological simplicity (standard time course correlations). For these reasons of computational efficiency and speed, PPC would seem to lend itself well to real-time implementation when compared to other CFC methods. Additionally, this mode of coupling may be more robust to noise due to its reliance on power (or amplitude) rather than phase, the latter of which may be more susceptible to signal noise (Giehl et al., 2021). For these reasons, the PPC metric was chosen as the mode of analysis.

In this study we focus on absence seizures because they are the most common type of childhood epilepsy and represent several challenges to clinicians. These challenges stem from the unique characteristics of absence seizures and their impact on the individuals who experience them. Absence seizures are often subtle and brief, lasting only a few seconds. The lack of convulsions or dramatic physical movements can make them less noticeable to observers, including clinicians. This subtlety may lead to under-recognition and misinterpretation of the seizures. Furthermore, the presentation of absence seizures can vary among individuals. Some may experience typical absences with staring spells, while others may exhibit more atypical features, such as subtle facial movements or eye fluttering. This variability makes diagnosis and recognition challenging for clinicians. The symptoms of absence seizures can also overlap with other neurological or psychiatric conditions. Clinicians must differentiate absence seizures from conditions like daydreaming, attention-deficit/hyperactivity disorder (ADHD), or other types of seizures. This requires a comprehensive clinical evaluation, including EEG monitoring.

Thus, while absence seizures are generally considered less severe than some other types of seizures, they present a range of challenges for clinicians, from the subtlety of their presentation to their potential impact on cognitive function and daily life. Accurate counting of absence seizures is crucial for optimizing therapy. Current diagnostics rely on clinical history, in-hospital video-EEG monitoring, and patient-maintained seizure diaries. However, research indicates that patients report only 6% of all experienced absences (Keilson et al., 1987), while caregivers report 14% (Akman et al., 2009). Therefore, a multipronged approach, including careful clinical evaluation and long-term EEG monitoring, is essential to address those challenges and to provide optimal care for individuals with absence seizures.

Scalp EEG is being used for long-term continuous monitoring with patients both in the intensive care units (ICU) and outside of the hospital. Patients may have spontaneous absence seizures that are easy to miss by the ICU staff or others, and long-term scalp EEG monitoring reduces the possibility of such oversight. Detecting seizures is critical for proper diagnostics and the increasing development of more portable and wearable EEG devices is making long-term monitoring of patients more practical and accurate.

Automated analysis is obviously important for real-time monitoring, and cutting-edge artificial intelligence techniques, particularly deep learning neural networks, offer robust tools for the automated analysis of EEG, including the exploration of cross-frequency coupling between distinct EEG rhythms. Deep learning stands out from other types of machine learning (ML) in that it is specialized for big datasets (including image matrices), complex features, and has superior ability to detect multifaceted latent patterns. For these reasons, it is not surprising that many successful classification studies have relied on various DL networks (Schirrmeister et al., 2017; Liu et al., 2022). This method is thus optimally suited for validating intricate cross-frequency coupling patterns for seizure classification. In this context, we introduce a Stacked Sparse Autoencoder (SSAE) specifically trained to identify absence seizure activity based on unique cross-frequency coupling patterns within scalp EEG.

## 2 Methods

EEG records (sampling frequency = 250 Hz) from the open source Temple University Hospital database [the TUSZ corpus, (Shah et al., 2018)] were used in the study. This dataset contains de-identified relatively short records of EEG from epilepsy patients of different ages with seizures annotated by neurologists (including both the seizure type as well as the start and stop times of the seizure). The dataset contained recordings that include 19 scalp EEG channels in accordance with the 10–20 configuration. The recordings’ sampling rate of 250 Hz allows for a range of high frequencies to be evaluated in the data (up to 120 Hz). The studies were conducted in accordance with the local legislation and institutional requirements and the relevant ethical guidelines and regulations, and was approved by the Georgetown-MedStar Institutional Review Board. All records with absence seizures available in the TUSZ corpus were taken into analysis. The total number of patients was 12. The annotations for each EEG record contained the seizure type (as determined both by EEG as well as clinical/behavioral characteristics) alongside the respective onset and offset times of that seizure. The duration of EEG records in the dataset varied from 5 to 35 min and the number of seizures in each record varied from one to 18. Although the TUSZ EEG records are not very long, they do represent continuous recordings which may include interictal, preictal and postictal activity. For the classification purposes, all segments containing only seizures (i.e., from the annotated onsets to the corresponding offsets) were cut from the initial records and used as the first data class comprising 94 seizure segments. Non-seizure segments (the second data class) were cut from the initial records such that they matched the number and the durations of seizure segments for each patient. The second class is referred to as ‘background’ activity. Thus, the overall dataset was balanced across two classes (the same number and the same duration of both seizure and background segments for each patient) with the average segment duration = 8.6 ± 5.3 s (mean ± standard deviation).

All EEG segments were taken into analysis as raw signals (i.e., without any preprocessing) in order to test the suitability of the current method to be applied to the raw EEG either online or offline. The analysis was performed using a modified script based on the PowPowCAT toolbox for EEGLAB (Thammasan and Miyakoshi, 2020). First, the spectrogram based on short-time Fourier transform was calculated for each EEG segment using the Matlab *spectrogram* function with half-overlapping one-second epochs and a Hamming window, for frequencies from 1 to 120 Hz (logarithmic scale: [1 1.28 1.56 1.85 … 19.1 19.8 20.6 … 110.6 113.7 116.8 120] Hz). The spectrogram provided the modulations of spectral power across time (within a given EEG segment) for each frequency, channel and segment. For each pair of frequencies and each EEG channel, power-to-power coupling was calculated as a Pearson correlation between the corresponding spectral-power time courses across a given EEG segment resulting in the channel-specific PPC matrices. Those matrices were averaged across all 19 EEG channels resulting in the CFC matrix for a given EEG segment.

The segment-specific PPC matrices (of size 100×100) were converted into the 4,950-point vectors by taking only the elements below the main diagonal (because PPC matrices are symmetrical around the main diagonal). These vectors were then used as training and testing sets for the Stacked Sparse Autoencoder (SSAE). The SSAE method begins by using unsupervised training to find the most characteristic features of the input classes and thus reduces the dimensionality of the inputs. This feature may be important to make the data analysis more robust against the intrinsic noise and individual variations of the EEG signal (see Results and Discussion below). The SSAE network was created with Matlab (v. R2023b) and consisted of two hidden encoder-decoder layers and the softmax layer with two outputs for binary classification ‘seizure vs. background’. The default (i.e., recommended by Matlab) values of the SSAE network internal hyper-parameters and structure were used which included L2 and sparsity regularizers. Regularizers are usually used to prevent overfitting of the network and increase its ability to generalize. L2 regularization adds the squared magnitude of coefficients to the loss function thus penalizing large weights while the sparsity constraint penalizes the loss function such that only a few neurons are active in a hidden layer. This helps the automatic detection of the most relevant features in the training sets. As a result of a preliminary exploration of the autoencoder with the given dataset, the optimal parameters of the hidden layers were found as achieving the stop condition during training (see Methods) in a shortest time (~6 min). The first encoder-decoder layer had 500 elements/neurons and the second layer had 50 elements/neurons. Thus, the reduction in input dimensionality by a factor of ~100 was achieved with two hidden layers.

A well-established approach ‘leave-one-subject-out’ was used for cross-validation purposes. For each subject, the SSAE training was performed using data from all other subjects and the selected subject’s segments (not used in training) were then tested and classified by the trained network. This approach eliminates bias in the results if the data from a single subject is included in both the train and test set and thus tests the model generalizability for data not used in training. The results from all subject-specific tests were then averaged across all subjects for the final values (mean ± standard deviation) of sensitivity, specificity and accuracy. To further evaluate the performance of the SSAE classifier, the receiver operating characteristic (ROC) curve as well as the precision-recall (PR) curve were calculated using Matlab function *rocmetrics*. As a result, the following parameters: AUC (area under the ROC curve), AUPRC (area under the PR curve) and the F1 score were derived.

## 3 Results

Among the 12 patients whose data were used in this study, there were 5 males and 7 females. The max/min age of the patients was 22/4 years and the average age was 10 ± 6.1 years. Demographic data of patients and clinical characteristics of their absence seizures are presented in Table 1. Half of patients had ‘atypical’ absence seizures due to their ‘focal’ features at the onset (for example, seizure activity predominantly at the frontal or temporal electrodes with rapid secondary generalization) or the presence of minor muscular phenomena (eye blinking or involuntary twitching).

**Table 1:** Demographic information and clinical features of patients’ absence seizures.

Subject #	Gender	Age	Clinical features of absence seizures
675	F	4, 6	Atypical absence seizure (blinking).
1113	F	20	Absence seizures.
1413	F	10, 14	3 to 6 Hz generalized spike and slow wave activity; seizures lasting 10–16 s.
1795	F	9	Atypical absence seizures. 3 to 5 Hz spike and slow wave activity preceded by symmetric focal (frontal) activity.
1984	M	6	Atypical absence associated with involuntary twitching and motion arrest.
2448	M	4	Typical of absence seizures but with a possibility of a secondarily generalized mechanism including the left frontal activity seen at the onset.
2657	M	5	Multiple absence seizures.
3053	F	5	EEG suggests more than one mechanism for seizures in this patient.
3281	M	13	The seizures were frontally predominant and relatively characteristic of absence epilepsy.
3306	F	13	Typical absence seizures.
3635	M	6	Generalized SW discharge with a clear underlying frontal focality.
8608	F	22	Atypical absence seizure with focal features (subtle focal slowing and sharp waves at T3, T5, and C3).

**Figure 1:**
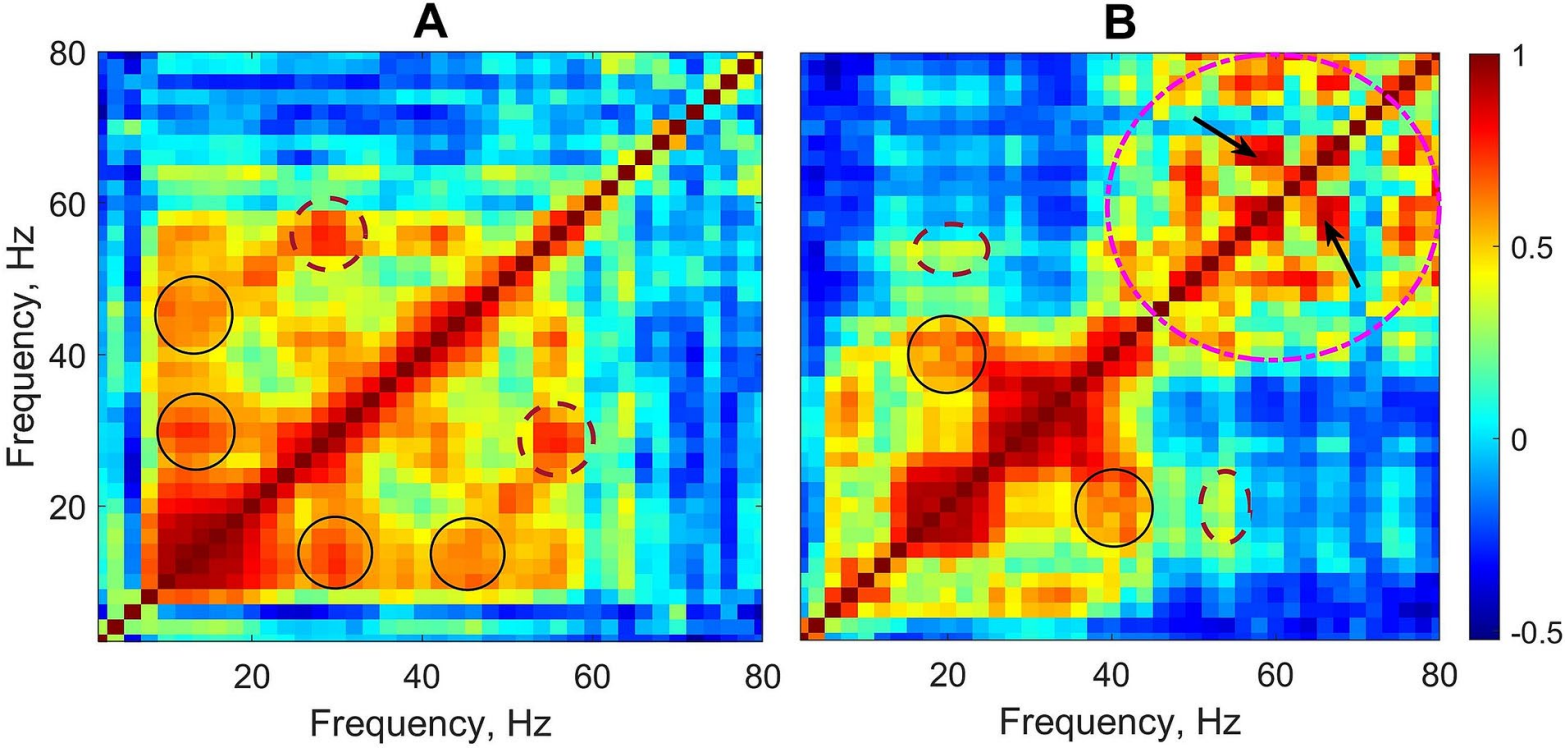
Two examples of cross-frequency matrices of EEG activity during absence seizures from two patients (linear frequency scale is used to demonstrate the arithmetic progression-like frequency relationships between harmonics). Circles, ovals and arrows show examples of a relatively stronger coupling between different frequencies including both harmonic and non-harmonic relations. See text for details.

Two typical examples of cross-frequency matrices for EEG activity during absence seizures taken from two different patients are shown in Figure 1. The overall pattern of the power-to-power frequency coupling was characterized by multiple discrete local maxima forming a ‘grid’ always symmetrical along the main diagonal. An approximately equal spacing between those maxima suggested that they reflected cross-frequency coupling between *harmonics*. Harmonics are integer multiples of the fundamental frequency arising in the spectral domain as a consequence of the main waveform not being strictly sinusoidal. Therefore, a relatively high coupling between the main frequency and its spectral harmonics is expected because harmonics occur at predictable intervals within the main waveform. For example, in Figure 1A some maxima (off the main diagonal) are present at the xy-coordinates of (15, 30) and (30, 15) Hz and (15, 45) and (45, 15) Hz (black solid circles). These maxima are likely to represent harmonics of the main frequency 15 Hz. Also, there are maxima at (28, 56) and (56, 28) Hz (brown dashed circles) which represent the first harmonic of frequency 28 Hz. Similarly, in Figure 1B there are maxima at the xy-coordinates of (20, 40) and (40, 20) Hz (black solid circles) which represent the first harmonic of the main frequency 20 Hz.

The cross-frequency patterns in the data, however, were not limited to the harmonics of the frequencies within the beta range. For example, Figure 1B also shows maxima at the coordinates of (20, 54) and (54, 20) Hz (the brown dashed ovals), and clearly the frequencies 20 Hz and 54 Hz are *not* harmonically related. Moreover, there are multiple maxima within the gamma band 40–80 Hz (the pink dashed circle) which demonstrate the coupling of gamma frequencies not harmonically related to each other (e.g., 58 and 66 Hz, arrows in Figure 1B).

**Figure 2:**
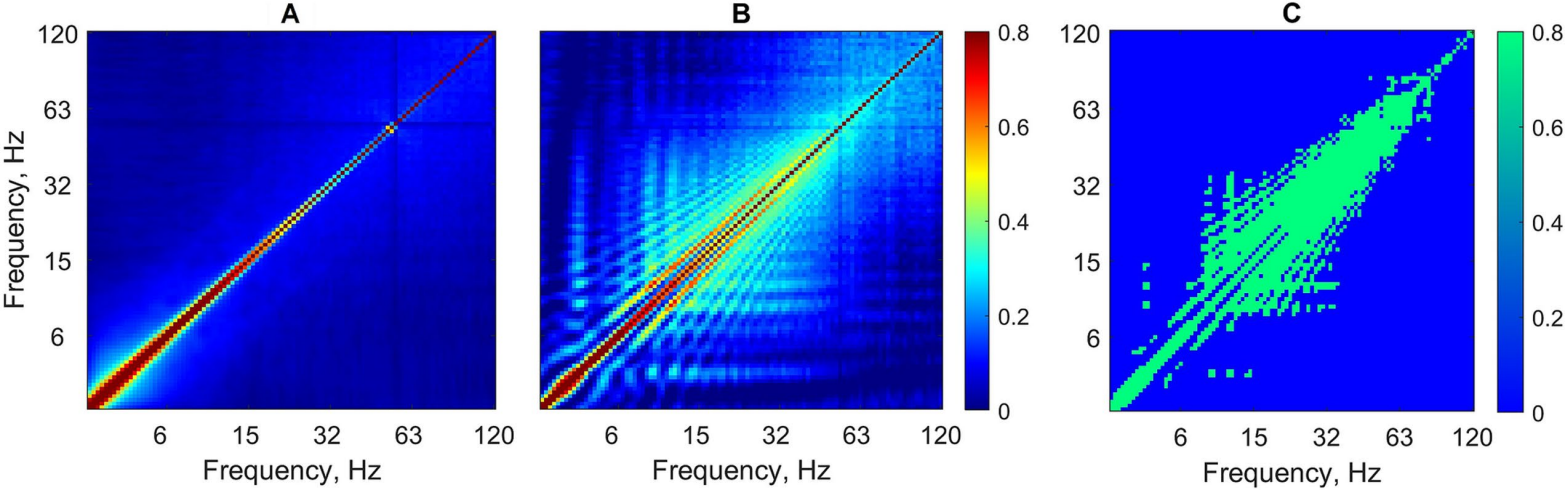
Cross-frequency coupling analysis without EEG preprocessing. Power-to-power matrices are group-averaged over all background segments **(A)** as well as absence seizures **(B)** (logarithmic frequency scale). **(C)** Statistically significant differences between two conditions for each frequency–frequency pair are shown in green (Mann–Whitney test, p < 0.05, FDR-corrected for multiple comparisons).

Cross-frequency coupling matrices group-averaged over all background as well as absence seizure EEG segments are shown in Figures 2A,B. Statistical testing for the difference between the two conditions (seizure versus background) showed that power-to-power coupling during seizures was significantly stronger for a wide range of frequencies from 6 to ~60–90 Hz (Figure 2C) (Mann–Whitney U-test, *p* < 0.05, FDR-corrected for multiple comparisons).

**Figure 3:**
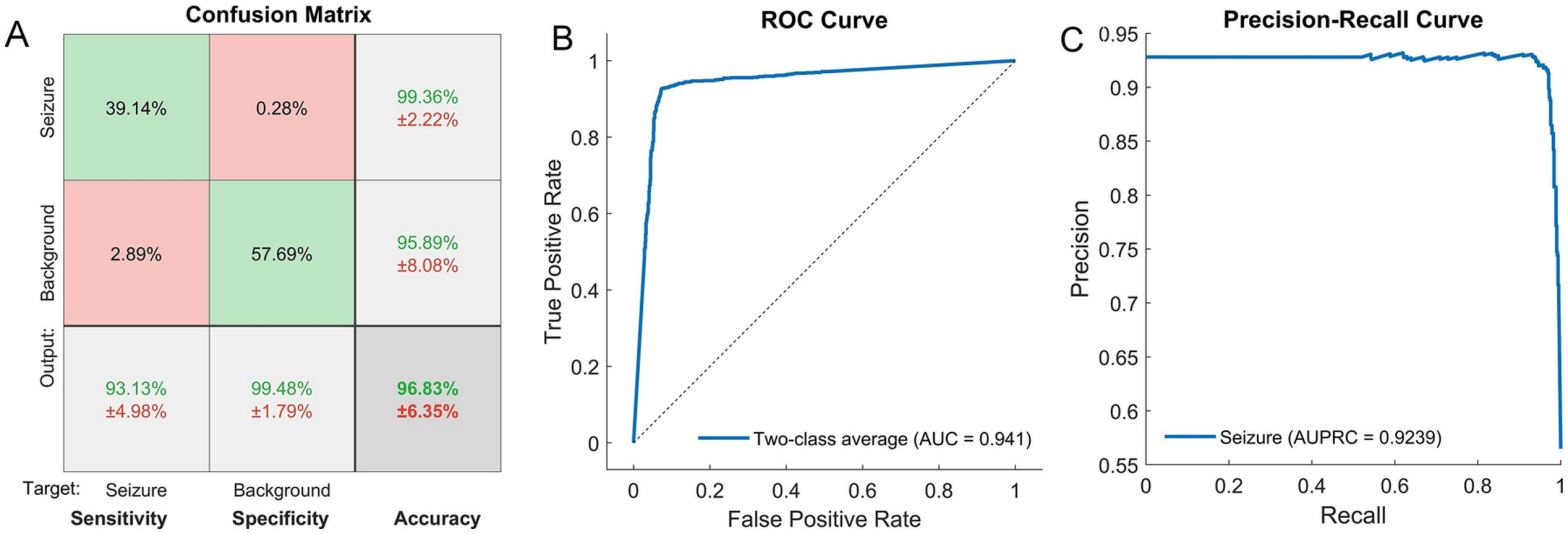
**(A)** Confusion matrix showing the results of recognition of seizures and background segments by the SSAE network. The mean values (%) ± standard deviations (%) are shown for sensitivity, specificity and overall accuracy (the bottom row) as well as for positive predictive values for each class (two upper cells in the right-hand column). (B, C) The results of two-class classification (seizure versus background) for the trained SSAE neural network. The ROC curve representing the classification results over both classes **(B)**. The precision-recall curve for the seizure class **(C)**. The corresponding metrics, i.e., the areas under the curves, AUC **(B)** and AUPRC **(C)**, are also shown.

During training, the network with L2 and sparsity regularizers achieved a squared error smaller than 10^−2^ (the stop condition) with about 400 iterations. After that, fine tuning was performed. Figure 3A shows the confusion matrix with the results of classifying seizures versus background segments by the SSAE network. On average, the trained network was able to correctly classify EEG segments (not used in training) at a sensitivity of 93.13%, a specificity of 99.48%, and an overall accuracy of 96.83%. Given the total duration of all EEG segments analyzed, the false positive rate of 0.52% = 100% - specificity; (Figure 3A) translates to 3.2 false alarms per hour.

The ROC and the PR curves are shown in Figures 3B,C with the corresponding values of the areas under the curve: AUC = 0.94 ± 0.057 (for the two-class average ROC), AUPRC = 0.92 ± 0.062 (for seizures), and the F1 score = 0.96 ± 0.046 (mean ± standard deviation).

Although the primary analysis of EEG records described above was purposefully done without conventional EEG preprocessing, in order to see whether preprocessing might improve the classification results, we repeated the same analysis after the following preprocessing steps: high-pass filtering at 0.1 Hz cutoff (a zero-phase FIR filter with the *filtfilt* function), notch filtering (filtering out the line frequency and its harmonics using the CleanLine EEGLAB toolbox), re-referencing to common average, Independent Component (IC) decomposition using the AMICA algorithm, and the automated removal of artifactual (‘bad’) ICs using the ICLabel algorithm (Pion-Tonachini et al., 2019). An IC was removed based on the following two criteria. First, if any of its probabilities (assigned by the ICLabel algorithm as a percentage) of being ‘muscle’, ‘eye’, ‘heart’, ‘line noise’ or ‘channel noise’ was greater than the probability of being ‘brain’ or ‘other’. Second, if the sum of the percentages of all the above artifactual assignments for this IC was greater than 50%.

The average CFC matrices after preprocessing are shown in Supplementary Figure S1, and, in comparison to the CFC matrices without preprocessing, they look very similar (compare Figure 2 and Supplementary Figure S1). EEG preprocessing slightly improved the classification of EEG segments (‘seizure versus background’) with the following results: *Sens* = 96.31%, *Spec* = 99.87%, and *Acc* = 98.51% (Supplementary Figure S2). However, all these classification metrics as well as the areas under the corresponding curves (the ROC curve and the precision-recall curve) were not significantly higher in comparison with the metrics obtained without preprocessing (Mann–Whitney test, *p* > 0.1 for all individual comparisons).

There is a question whether cross-frequency coupling, as an EEG feature, presents any advantage compared to the spectral power used in many published classification approaches. To address this question, we ran an additional analysis using spectral power for classification purposes. For each EEG segment, the output of the PowPowCAT function also provided power spectra (i.e., the spectrogram averaged across time). Similar to the PPC matrices, the channel-specific spectra were averaged across channels and the resulting segment-specific spectra were used as an input to the SSAE (Supplementary Figure S3, *left*). Classification based on the power spectrum produced slightly worse but non-significantly different results compared to the classification based on PPC: 90.1 ± 21 vs. 93.1 ± 4.98; 98.4 ± 5 vs. 99.5 ± 1.8; 95.5 ± 10 vs. 96.8 ± 6.4 (%, mean ± st. dev.; for *Sens*, *Spec*, and *Acc*, respectively) (Supplementary Figure S3, *right*). Importantly, however, the standard deviations for the spectrum-based classification metrics were significantly larger compared to the PPC-based classification (Bartlett’s test; Table 2). The larger variance of the spectrum-based classification results was likely due to the individual differences in spectral characteristics of the EEG. Also, this result indicates that cross-frequency coupling may provide an EEG feature which is more robust against the individual variations.

**Table 2:** Performance comparison of the PPC-SSAE classifier with other classifiers.

	Sensitivity		Specificity		Accuracy	
	Mean, %	St. Dev., %	Mean, %	St. Dev., %	Mean, %	St. Dev., %
PPC-SSAE	93.1	5.0	99.5	1.8	96.8	6.4
Spectra-SSAE	90.1	21.0***	98.4	5.0**	95.5	10.0*
PPC-SVM	81.1	32.0**	97.1	14.0***	90.0*	16.0**
PPC-Random Forest	78.0*	24.6***	91.5**	13.2***	85.6***	11.6**

PPC, power-to-power coupling; SSAE, stacked sparse autoencoder; SVM, support vector machine. PPC-SSAE, algorithm based on PPC as a classifying feature and the stacked sparse autoencoder as a classifier; Spectra-SSAE, algorithm based on the EEG spectrum as a classifying feature and the SSAE classifier; PPC-SVM, algorithm based on PPC as a classifying feature and the SVM classifier; PPC-Random Forest, algorithm based on PPC as a classifying feature and the Random Forest classifier. The PPC-SSAE algorithm is compared to all three other algorithms with statistically significant differences indicated by asterisks: *p* < 0.05 (*), *p* < 0.01 (**) and *p* < 0.001 (***) (*t*-test for the comparison of the mean values, Bonferroni-corrected; Bartlett’s test for the comparison of variances, Matlab function vartestn).

To compare the performance of the SSAE-based classifier with the ML algorithms, we used the Support Vector Machine (SVM) and the Random Forest (RF) classifiers using the same PPC matrices as input and the same ‘leave-one-subject-out’ cross-validation approach. Matlab functions *fitclinear* and *fitensemble* were used for the SVM and RF classifiers, respectively. For each subject ‘left-out’, the SVM training was performed using 5-fold cross-validation on the remaining subjects with the subsequent testing of the excluded subject. For the RF classifier, the number of trees varied from 1 to 250 with the control of the out-of-bag error. It appeared that the error leveled out in the range of 20–140 trees and remained at the lowest value thereafter, insignificantly affecting the classification accuracy. After the preliminary testing, the RF-based classification was performed with number of trees = 140 with the same ‘leave-one-subject-out’ procedure. Both SVM and RF classifiers performed worse than the SSAE classifier with lower values of *Sens*, *Spec* and *Acc* as well as a significantly greater variance of those metrics (Table 2).

## 4 Discussion

Ongoing research in machine learning and deep learning is actively exploring absence seizures to identify their critical features, aiming to gain deeper insights into the electrophysiological roles that these features play, with the goal of improving seizure detection and prediction. In both murine and human studies, successful training of the networks usually involves using relevant time and frequency domain metrics especially frequency and amplitude, and sometimes phase (Fanselow et al., 2000; Xanthopoulos et al., 2009; Richard et al., 2015; Kumar et al., 2021). Most studies use wavelet analysis techniques to account for non-stationarity of the EEG signal and improve time and frequency localization of various EEG patterns. Entropy-related metrics, especially permutation entropy (PE), was also very useful in training networks, and decreases in PE were found in both preictal and ictal segments in comparison to background (Li et al., 2007). Furthermore, specific spatial features were found to characterize absence seizures such as increased cortico-thalamo-cortical synchrony in murine models, or reductions in overall functional connectivity patterns during generalized spike-and-wave discharges in humans (van Luijtelaar et al., 2016; Kumar et al., 2021).

Many studies found that the harmonics of the fundamental frequencies of seizures are highly specific and critical to the classification success (Sitnikova et al., 2009; Buteneers et al., 2013). Harmonic spectral analysis involves broad wavebands (i.e., 1–120 Hz) that include HFOs which are increasingly recognized as crucially important in the pathophysiology of epilepsy. The energy in these higher frequency harmonics are found to be important signatures differentiating between regular sleep spindles, artifacts and true spike-and-wave discharges that all share the same fundamental frequency (Sitnikova et al., 2009). The interdependent and harmonic architecture of the EEG frequency spectrum has been well described by authors such as Buzsáki Buzsaki and Draguhn (2004) and Klimesch (2013) and indicates that a comprehensive analysis of EEG activity should involve a view of the cross-frequency dynamics.

### 4.1 Comparison with other machine learning and deep learning methods

The use of ML algorithms and DL neural networks in studies attempting to recognize and predict absence seizure EEG activity has been rapidly advancing in the past decades, generating promise in improving both clinical treatment as well as the neurobiological understanding of this disorder. Studies since the early 1990’s describe the ability of ML and DL methods to recognize absence seizures with high level of sensitivity (~95%) albeit often with higher rates of false positives (Jando et al., 1993; Vadasz et al., 1995). Many of these earlier studies used genetic murine models of absence epilepsy and implanted EEG electrodes. More recent ones apply these techniques to humans using only scalp EEG and with the ability to run the computation not only offline, but also in real time (Alam et al., 2024).

This ability to differentiate the pre-seizure from the seizure state is now being successfully applied to humans using scalp EEG with as few as 19 scalp electrodes (Kumar et al., 2021). Schirrmeister et al. (2017) used spectral power between alpha-high gamma bands with a convolutional neural network (CNN) and achieved accuracies as high as 84% (Schirrmeister et al., 2017). In a more recent exploration with a shallow CNN applied to scalp EEG data from human subjects, Zhang et al. achieved a sensitivity of 92.2% with a low false positives rate (FPR) of 0.12 per hour (Zhang et al., 2020). Other studies too have used various ML and DL models for seizure detection and/or prediction in human scalp EEG using different features with accuracy ranging from ~70% to higher than 90–95% (Li et al., 2016; Sridevi et al., 2019; Ansari et al., 2021; Liu et al., 2022; Thara et al., 2023; Alam et al., 2024).

The current SSAE-based classification results are on par with or better than several studies based on other DL neural network classifiers such as: CNN and BiLSTM (Liu et al., 2022; Schirrmeister et al., 2017; Zhang et al., 2020; Thara et al., 2023; Khan et al., 2022), DCNN (Fujita et al., 2022), multiple neural networks (not specified) (Yang et al., 2023) (see Table 3). We are aware of only one study based on a convolutional neural network which reported 100% for sensitivity, specificity and accuracy (Akut, 2019). However, this study was done on a dataset of only 5 patients which raises a question about the generalizability of this result. A comprehensive comparison of various CNN networks, including pretrained GoogLeNet and AlexNet as well as the authors’ original hybrid model (AG86), was done in Khan et al. (2022). Their hybrid model AG86 combined the best features of GoogLeNet (inception layer) and AlexNet (starting and ending layers) and demonstrated a better performance than several other pretrained networks (Khan et al., 2022). Although the proposed SSAE classifier showed a slightly lower sensitivity (93%) compared to the AG86 model (95%), it achieved better specificity (99%) and accuracy (97%) (Table 3). Also, the SSAE classifiers usually have just two hidden layers and thus have a simpler architecture compared to the CNN networks which require multiple hidden layers to achieve a comparable reduction in dimensionality (Akut, 2019), and this requires more computational resources. For example, in Akut’s study the training was done on Tesla K80 GPU to achieve faster computation time. The GPU used 12 GB Memory, 61 GB RAM and 100 GB SSD (Akut, 2019). In comparison, the proposed SSAE classifier was realized on a laptop (with the Windows 10 Enterprise OS) with Intel(R) Core(TM) i5-5300U CPU at 2.30GHz, 16 GB RAM and ~ 1 GB hard drive space. This speaks to an excellent computational efficiency of the SSAE classifier. EEG classification using SSAE is a novel approach and we are aware of only one study where a SSAE classifier for seizure detection also demonstrated very good performance (*Sens* = 93%÷100%; *Spec* = 90%÷100% and *Acc* = 96%) (Lin et al., 2016).

**Table 3:** Seizure classification/detection studies using deep learning neural networks.

Author and year	Feature	Classifier	*Sens*	*Spec*	*Acc*	Dataset	Fs, Hz	No. of subjects	Subject-specific algorithm
Lin et al. (2016)	Raw data	SSAE	93–100%	90–100%	96%	Bonn	173	5	No
Schirrmeister et al. (2017)	Raw data	CNN	-	-	84%	TUH	250	14	Yes
Akut (2019)	Raw data	CNN	100%	100%	100%	Bonn	173	5	No
Zhang et al. (2020)	Raw data	Shallow CNN	92%	-	-	CHB-MIT	256	-	No
Liu et al. (2022)	Raw data	CNN, BiLSTM	86%, 89%	-	97.5%93.7%	CHB-MIT, SH-SDU	256	33	No
Fujita et al. (2022)	PAC	DCNN	90%	90%	90%	Innov. AI Hosp.	1000–2000	180	No
Khan et al. (2022)	Scalogram	Various CNNs	95%	95%	95%	TUH	250	9	No
Yang et al. (2023)	Temporal spectral	Multiple NNs	98%	100%	85%	TUH	250	14	No
Thara et al. (2023)	Raw data	CNN, VGGNet, ResNet	-	-	97%	TUH	250	-	No
This work	PPC	SSAE	93%	99%	97%	TUH	250	12	No

*Sens*, sensitivity; *Spec*, specificity; *Acc*, accuracy; Fs, sampling frequency, hyphen (−), no data.

It is also important to compare the DL-based models with more traditional ML algorithms. Since 2012, emerging research in epilepsy classification utilizing ML has shown dramatic improvements in sensitivity, specificity, and/or accuracy (up to 100% sensitivity). For example, one of these studies used increasingly larger datasets than previous studies such as with the number of patients up to 23 (Chandel et al., 2016), and still obtaining a sensitivity of 100%. It is worth noting that the ML methods typically outperformed DL neural networks in seizure classification on certain datasets achieving sensitivity at 100% as well as specificity and accuracy at 99% (Saeed et al., 2016; Chandel et al., 2016; Khan et al., 2012; Ansari et al., 2021) (see Tables 3, 4). Moreover, the ML algorithms are more compact and allow an effective implementation in hardware (Alam et al., 2024). However, more recent research on DL has shown similar capabilities (Khan et al., 2022; Thara et al., 2023; Akut, 2019), and more research with DL is warranted. The DL-based classification algorithms can continue to improve by broadening their approach to patient-independent training, including larger datasets with more patients, and optimizing sensitivity, specificity and accuracy. In the current study, the performance of the SVM and Random Forest classifiers were significantly lower compared with the SSAE classifier and lower than the reported results for the ML classifiers in many other studies (see Tables 2, 4). It is likely that the more modest results with the SVM and RF classifiers were due to a more stringent ‘leave-one-subject-out’ cross-validation used in the current study. Also, the larger variance of the classification metrics (*Sens*, *Spec*, and *Acc*) with the SVM and Random Forest algorithms point to the lower generalizability of the ML classifiers compared to the SSAE classifier in the current study.

**Table 4:** Seizure classification/detection studies using machine learning algorithms.

Author and year	Feature	Classifier	*Sens*	*Spec*	*Acc*	Dataset	Fs, Hz	No. of subjects	Subject-specific algorithm
Khan et al. (2012)	Skewness kurtosis	Simple linear classifier	100%	-	-	CHB-MIT	256	10	Yes
Li et al. (2016)	Entropy	LDA	-	-	89.0%	Peking Univ. Hosp.	256	10	No
Saeed et al. (2016)	Entropy, CSD	SVM	100%	-	-	CHB-MIT	256	10	Yes
Chandel et al. (2016)	Spectral, entropy	Linear classifier	100%	-	-	CHB-MIT	256	23	Yes
Jacobs et al. (2018)	PAC	Random Forest	87.9–97.5%	82.4–95%	80–95%	Toronto Western Hosp.	500–1,024	12	Both
Liu et al. (2018)	PAC	SVM	-	-	97.5–100%	CHB-MIT, Bonn	256, 173	28	-
Sridevi et al. (2019)	Spectral, entropy	LDA, NB, DT, SVM, KNN	80%	86%	83%	SCTIMST, Fortis Malar Hosp.	256–400	18	No
Ansari et al. (2021)	Spectral	Linear classifier	100%	99%	99%	AIIMS, CHB-MIT	128–256	30	No
Alam et al. (2024)	Various	QDA classifier	100%	-	99.4%	Bonn	173	5	No

*Sens*, sensitivity; *Spec*, specificity; *Acc*, accuracy; Fs, sampling frequency, hyphen (−), no data.

Growing interest in using DL for seizure classification is partly related to how its unique characteristics may allow for increased generalizability including across individuals, seizure types, sleep vs. wake conditions, and eventually moving from dual classification (e.g., seizure vs. background) to ternary classification (e.g., seizure vs. preictal vs. background). The ability of the DL networks to eliminate or at least reduce the need for feature extraction may be part of this generalization. Getting rid of the human bias on what features define a seizure may improve seizure classification. In place of the ‘extracted-by-a-human’ features, DL has an ability to find more abstract and higher-level representations (Akut, 2019). DL’s greater number of hidden layers alongside nonlinear activation functions expand its abilities for finding intricate and nonlinear patterns in the data. Given that the brain is non-linear and its EEG signals are non-stationary and complex, it seems appropriate to continue to evaluate whether and how DL may match or improve traditional ML accuracy in seizure classification.

Our method has built on the efficacious components of the existing research in regard to the deep learning techniques, significant CFC biomarkers, and the emerging relationships of HFOs to epilepsy. The novelty and significance of this approach includes validation of a hitherto unexplored phenomenon of cross-frequency interactions (specifically, power-to-power coupling) in the context of identifying new biomarkers of absence seizures. Building on prior research which suggests the key importance of HFOs in epilepsy, this approach also holds promise for clinical application in long-term monitoring of patients with absence seizures.

It is becoming clear that there is a complex interplay between spectral, harmonic and spatial features that can reliably characterize absence epilepsy. PPC analysis has a level of sensitivity to these features already known to have utility in seizure classification (i.e., spectral power), and in addition, it provides important information on cross-frequency interaction. In this way, PPC represents a novel powerful, hitherto underutilized, tool to probe the unique cross-frequency signatures of epileptiform activity. It holds promise for further enhancing the optimization between sensitivity and specificity. This becomes particularly crucial in scenarios where data is less pristine or encompassing multiple states such as sleep and wakefulness. The results not only confirm the utility of a new approach to classify absence seizures with high accuracy, but also strongly suggest that continuing research on cross-frequency coupling will deepen our knowledge of the underpinnings of epileptic seizures by further clarifying the involvement of HFOs (which are already known to be deeply related to epilepsy), harmonic patterns, as well as interdependent relationships between different frequency bands more generally.

## 5 Limitations of the study

A limitation of the current study is the use of a single public dataset (TUSZ) which has EEG records of absence seizures from just 12 patients. Also, the available EEG records are not very long (5 to 35 min in duration). While the number of patients (12) is comparable to other classification studies (Table 3; with the exception of Chandel et al., 2016; Ansari et al., 2021; Fujita et al., 2022), this may still limit the generalizability of our results. However, the absence seizure dataset from the TUSZ corpus is relatively balanced by the gender of patients (7 females and 5 males) and it also contains a wide range of patient ages, from pediatric to young adult (4–22 years). The ‘one-subject-out’ cross-validation did demonstrate good generalizability across this range of patients’ ages.

Another limitation is that it is unclear whether the classification performance in the present study is achieved due to a specific feature set (i.e., power-to-power coupling matrices) or a specific classifier type (i.e., the autoencoder). However, the use of another feature namely, power spectrum, which has been used in many other studies, (e.g., Ansari et al., 2021; Yang et al., 2023), did not improve classification. Importantly, the PPC-based classification had significantly smaller variance compared to the spectrum-based one.

## 6 Conclusion

The results provide evidence both for the parameters of power-to-power coupling having utility for seizure classification and also for an approach using PPC alongside SSAE neural networks being efficacious for automated classification of seizures within scalp EEG. Importantly, the trained SSAE network showed generalizability in detecting seizures with high sensitivity (93%), very high specificity (99.5%) and accuracy higher than 96% with all patients tested. Automated analysis based on deep learning networks can significantly accelerate the analysis of EEG data and increase their diagnostic value.

## Data Availability

Publicly available datasets were analyzed in this study. This data can be found at: Temple University Hospital database (the TUSZ corpus) https://isip.piconepress.com/projects/tuh_eeg.
