# Supplementary material for: The classification of absence seizures using power-to-power cross-frequency coupling analysis with a deep learning network
Source: Front Neuroinform. 2025 Feb 10;19:1513661. doi: 10.3389/fninf.2025.1513661 (PMC11847813; doi:10.3389/fninf.2025.1513661)
Supplement: Supplementary file 1 [file Table_1.DOCX]

***Supplementary Material***

# Supplementary Figures

**
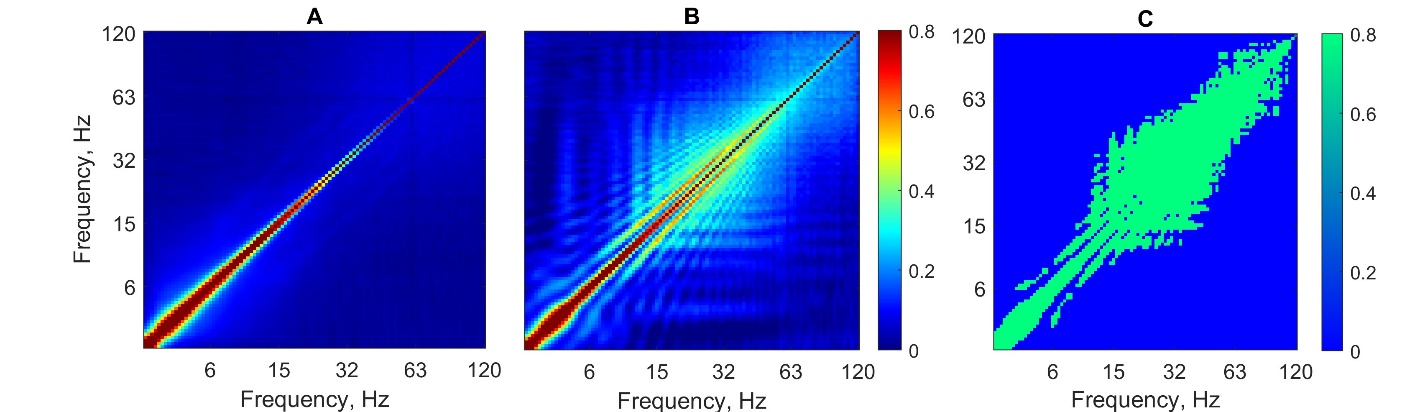
**

**Supplementary Figure 1.** Cross-frequency coupling analysis after EEG preprocessing. CFC matrices group-averaged over all background segments (*A*) as well as absence seizures (*B*) (logarithmic frequency scale) after preprocessing. (*C*) Statistically significant differences between two conditions for each frequency-frequency pair are shown in green (Mann-Whitney test, p < 0.05, FDR-corrected for multiple comparisons)

**
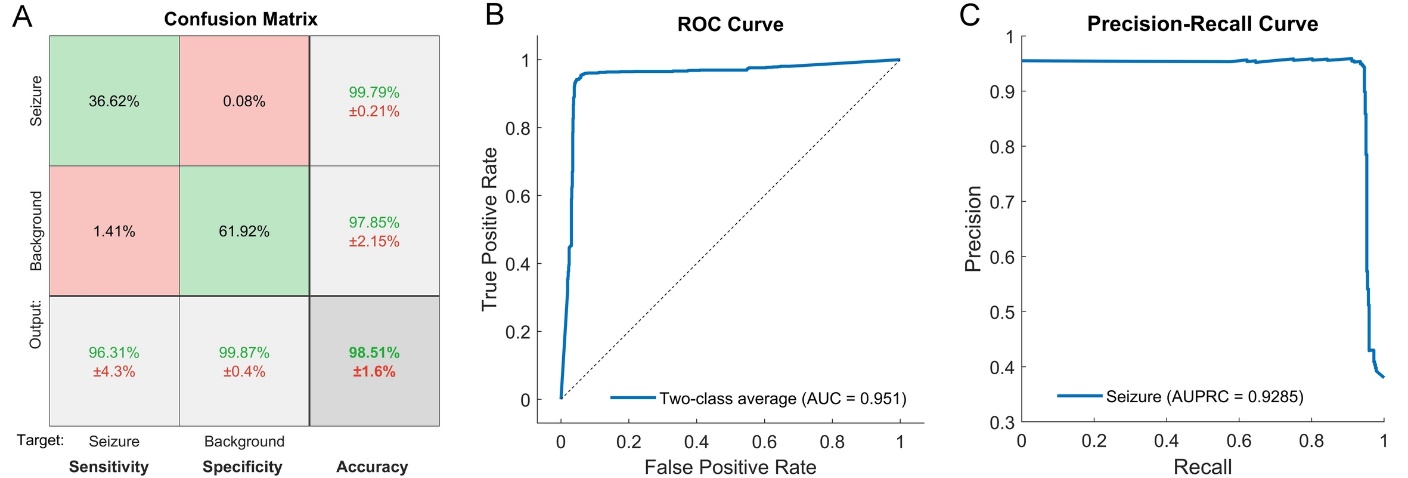
**

**Supplementary Figure 2.** The results of the classification ‘seizure versus background’ after EEG preprocessing. (*A*) Confusion matrix showing the results of recognition of seizures and background segments by the SSAE network. The mean values (%) ± standard deviations (%) are shown for sensitivity, specificity and overall accuracy (the bottom row) as well as for positive predictive values for each class (two upper cells in the right-hand column). (*B, C*) The results of two-class classification (seizure versus background) for the trained SSAE neural network. The ROC curve representing the classification results over both classes (*B*). The precision-recall curve for the seizure class (*C*). The corresponding metrics, i.e., the areas under the curves, AUC (*B*) and AUPRC (*C*), are also shown.

**
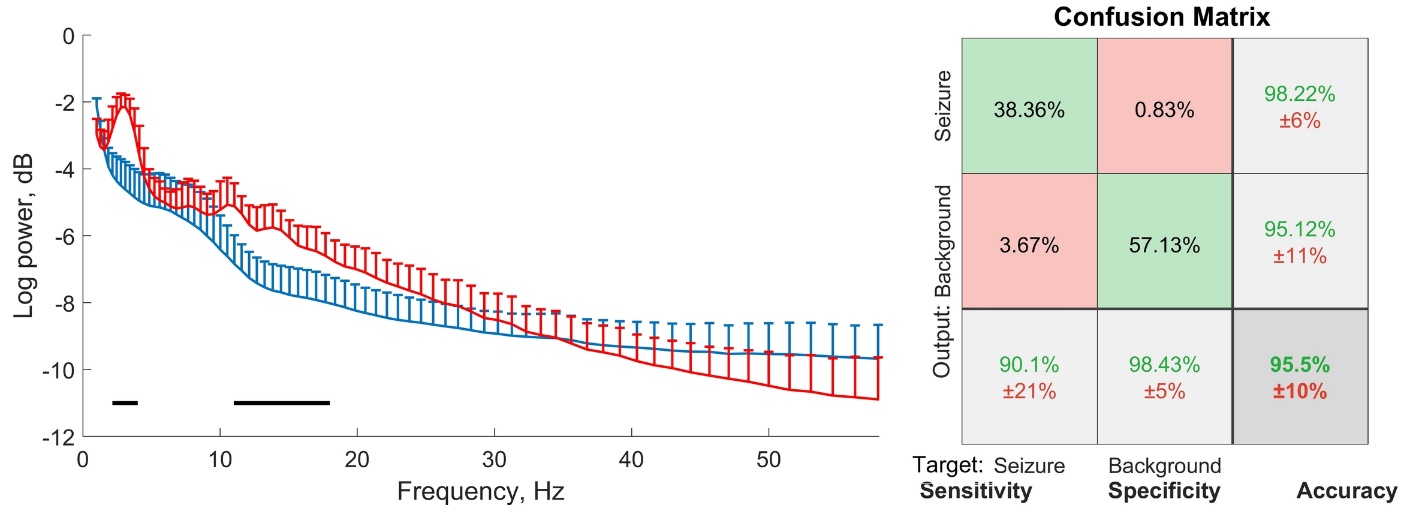
**

**Supplementary Figure 3.** (*Left*) Power spectra averaged over all channels and all segments for each data class: background (blue) and absence seizure (red). Standard deviations of the mean spectra are indicated by error bars. Horizontal black lines indicate frequencies at which the spectra were significantly different between two data classes (Mann-Whitney test, p < 0.05, FDR-corrected for multiple comparisons). (*Right*) Confusion matrix showing the results of recognition of seizures and background segments by the SSAE network, based on power spectra. The mean values (%) ± standard deviations (%) are shown for sensitivity, specificity and overall accuracy (the bottom row) as well as for positive predictive values for each class (two upper cells in the right-hand column).

| 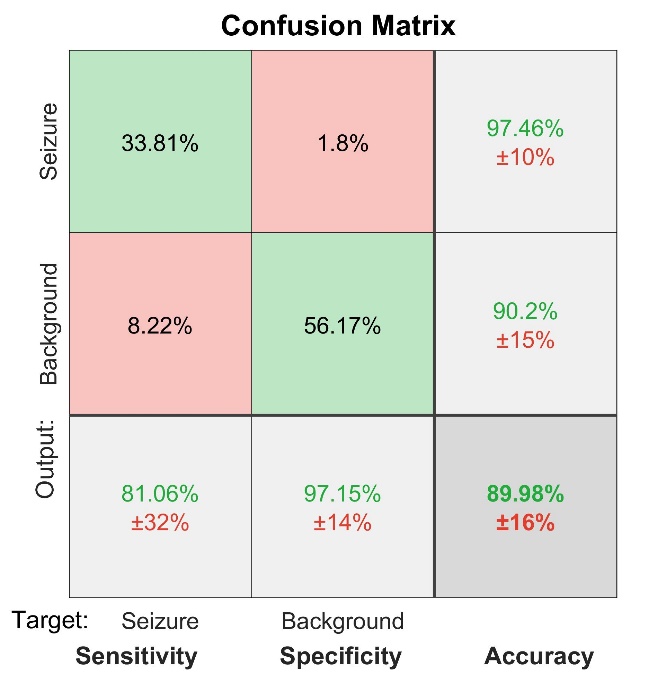 | 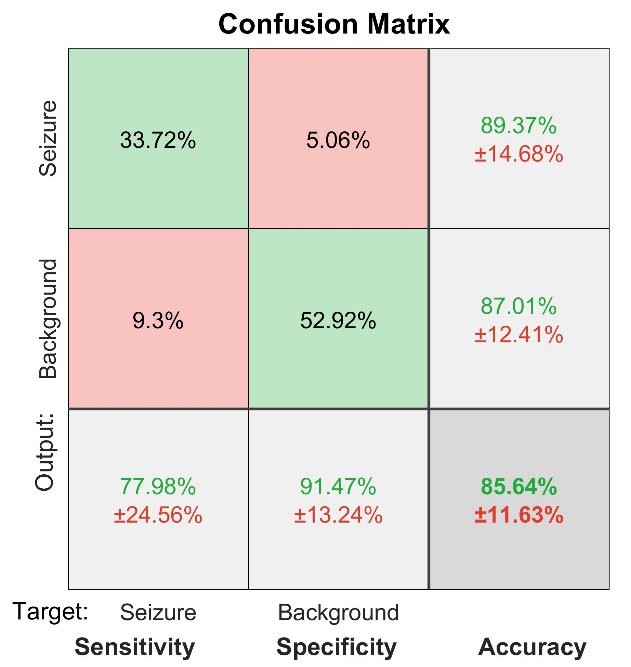 |
| --- | --- |

**Supplementary Figure 4.** Confusion Matrices showing the results of classification (based on power-to-power coupling matrices) of absence seizures versus background segments by the SVM (left) and Random Forest (right) algorithms. The mean values (%) ± standard deviations (%) are shown for sensitivity, specificity and overall accuracy (the bottom row) as well as for positive predictive values for each class (two upper cells in the right-hand column).
